# A Comprehensive Review of Congenital Lumbar Synostosis and Associated Findings

**DOI:** 10.7759/cureus.19013

**Published:** 2021-10-24

**Authors:** Albert M Volk, Mansour Mathkour, Joe Iwanaga, Aaron S Dumont, R. Shane Tubbs

**Affiliations:** 1 Anatomy, Tulane University School of Medicine, New Orleans, USA; 2 Neurosurgery, Tulane University School of Medicine & Ochsner Medical Center for Children, New Orleans, USA; 3 Neurosurgery, Tulane University School of Medicine, New Orleans, USA; 4 Neurosurgery, Ochsner Neurosciences Institute, Covington, USA

**Keywords:** congenital vertebral synostosis, vertebral segmentation, lumbar synostosis, klippel-feil syndrome, spine

## Abstract

Congenital vertebral synostosis (CVS) is a rare developmental condition due to failure of vertebral segmentation. Vertebrae and their intervertebral discs differentiate and resegment at the time of organogenesis during fetal life. Failure of this embryological process can result in the limitation of mobility of the involved segment. This inappropriate segmentation thus results in vertebral fusion or a block vertebra with subsequent vertebral synostosis. Long-term, such segmental fusion can increase osteoarthritis at levels below and above the fused segment due to excessive wear on these joints. Presentations can include congenital kyphosis and scoliosis. Patients may present with back and radicular pain, and possible myelopathy CVS usually occurs, in order of frequency, in the cervical, lumbar, and thoracic vertebral levels. This paper reviews congenital lumbar synostosis with associated findings and its clinical implications and embryological significance. A case illustration is also included.

## Introduction and background

Congenital vertebral synostosis (CVS) is a rare developmental condition due to failure in the process of segmentation or metamerism, which usually occurs (in order of frequency) in the cervical, lumbar, and thoracic vertebral levels. Vertebrae and their intervertebral discs differentiate and resegment at the time of organogenesis during fetal life [1,2]. Resegmentation is the hallmark of vertebral development and involves metamerism, whereby vertebrae can be fused completely, both anterior and posterior elements being involved, or partially [1,3,4]. Inappropriate vertebral fusion results in block vertebra or vertebral synostosis. Although there is continuous trabeculation through the fused segment, a remnant of the intervertebral disc is commonly present [3,4].

CVS can be isolated or result from syndromic manifestations such as Klippel-Feil syndrome (KFS), which causes spine deformity. Acquired vertebral synostosis (AVS) is due to an underlying pathology such as fibrodysplasia or progressive juvenile rheumatoid arthritis, follows an infection such as tuberculosis, or is posttraumatic or postsurgical [5,6]. CVS is usually asymptomatic and is found incidentally; however, in some cases, it presents with neck and back pain owing to alteration and stress of the spinal biomechanics leading to premature adjacent level degeneration, spinal canal stenosis and spine deformity [2]. This paper reviews congenital lumbar synostosis with associated findings and its clinical implications and embryological significance.

## Review

Embryology, timing and complete versus partial CVS 

The vertebral column derives primarily from the notochord and the paraxial mesoderm, which segments into somites during weeks 3-5 of gestation to form the future levels of the spine. The timing of spine development also explains the associated renal, cardiac, neuronal, musculoskeletal and other anomalies [7,8]. Each somite's sclerotomal component subdivides into cranial and caudal halves, which form the vertebral body, while the intervening spaces ultimately become the intervertebral disc. The sclerotome then migrates bilaterally around the neural tube to form the pedicles, laminae, and spinous processes [7]. Ablation studies have demonstrated that both the notochord and neural tube are essential for proper segmentation of the sclerotomes [9]. Cases in which the intervertebral disc fails to form and subsequent sclerotomal migration also fails can lead to CVS, which are estimated to occur in 0.5-1.0 per 1000 individuals [10]. Furthermore, right and left segments can progress independently, leading to asymmetric malformation. Even though CVS is commonly referred to as fusions, they are more accurately described as developmental unions between unseparated sclerotome segments [11]. 

The anatomy of complete versus partial fusion involves the timing of union during fetal development. Completely fused vertebrae and absence of articular facets suggest a failure of normal development and differentiation of vertebrae during the pre-cartilaginous stage [12], while partial union with independent pedicle and transverse process and a ridge on the dorsal surface of fused arches indicates normal initial development followed by synostosis. 

Clinical presentation / Association

While CVS is usually asymptotic and found incidentally, it can have minimal functional consequences with a notable fraction leading to clinically relevant pathologies, including congenital kyphosis and scoliosis [13,14] or stenosis of the neural canal with back and radicular pain and possible myelopathy [11,15] owing to alteration and stress of the spinal biomechanics resulting in premature degeneration of adjacent levels [16].

Congenital vertebral synostosis versus acquired vertebral synostosis

Radiographically, congenitally fused vertebrae exhibit the following: The intervertebral foramina involved become ovoid, and their diameters can be normal, larger, or smaller as in our case. The anteroposterior diameter of the vertebral body is often diminished, resulting in a "wasp waist" appearance on lateral images, often seen at the intervertebral disc level between the fused segments, which is usually absent in acquired synostosis [3,4]. However, the associated intervertebral disc is rudimentary or smaller than normal. As seen in our case, the associated facet joints are fused in up to half of all patients. The spinous processes can be malformed or fused [3]. Finally, the combined height of the block vertebra fused segments can be equal to or greater than the combined height of the involved bodies and their associated disc; those dimensions are less in acquired synostosis [3,4].

Case Presentation 

**Figure 1:**
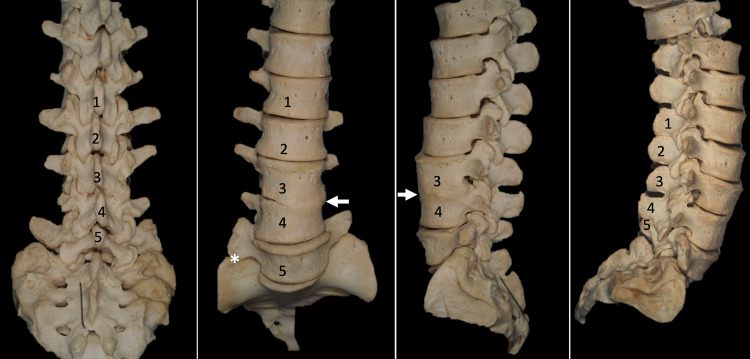
Photographs of the CVS presented herein, posterior, anterior, left lateral, and right lateral views. In the second panel, note the variant lateral articulation between the sacrum and L5 vertebra (*). Source: Images of skeletons preserved at the Tulane University School of Medicine

**Figure 2:**
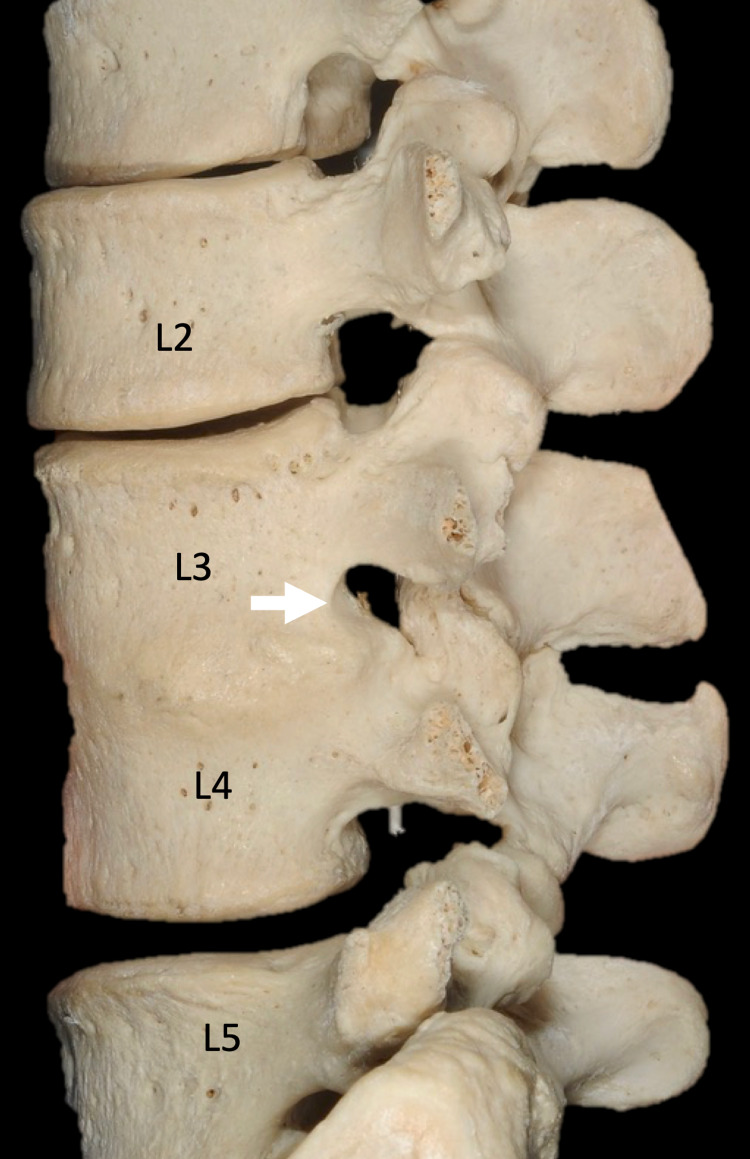
Zoomed in view of the left lateral view noting the degree of intervertebral foramina stenosis at L3/L4 and the anterior fusion of the bodies of these vertebrae (arrow) Source: Images of skeletons preserved at the Tulane University School of Medicine.

Congenital fusion of the L3 and L4 vertebrae is shown in an adult female spine (Figure 1, 2). This specimen was from our university's osteological teaching collection. Posteriorly, the facet joints, laminae, and anterior parts of the spinous processes are fused at this level. No segmental anomalies such as sacralization are found. On the right side, the transverse process of L5 forms a distinct articulation with the ala of the sacrum. Anteriorly, the anterolateral aspect of the bodies of L3 and L4 are fused. The intervertebral foramen at the L3/L4 level is 5 x 7mm in diameter on the left and 10 x 5mm in diameter on the right. The spinal canal at these two levels is stenotic. No other bony anatomical variations such as spina bifida are noted, and the remaining spine is within normal limits.

Syndromic association and clinical implications 

CVS can be isolated or can result from syndromic manifestations such as KFS, MURC (Mullerian duct aplasia, renal aplasia, cervicothoracic somite dysplasia), Escobar (autosomal recessive cervical vertebral fusion, ptosis, hypertelorism of neck, axillae, genital anomalies, and small stature), VACTERL (vertebral, anal, cardiovascular, tracheoesophageal, renal and limb abnormalities or single umbilical artery), hemifacial macrosomia, diabetic embryopathy, segmentation syndrome 1 with laryngeal malformation, Trisomy 18, Alagille, Joubert, and Jarcho-Levin syndromes [1,8]. KFS is on the locus Chromosome 8q and is associated with inversion inv [15] (q22.2 q23.3), which is associated and segregated with congenital vertebral fusion [5]. There are currently two main classification systems for patients with KFS: the original KFS subtypes (I, II, and III) and the more recent KFS classes (KF1-4). 

The original classification system, described by Maurice Klippel and Andre Feil in 1912, focused on cervical fusion. Patients with KFS type I present with an extensive fusion of the cervical and upper thoracic vertebrae. KFS type II is defined by a fusion of one or two cervical or thoracic vertebrae. Finally, KFS type III is characterized by a fusion of the cervical and the thoracic or lumbar vertebrae [17].

The more recent classification described by Clarke and colleagues distinguishes individuals based on three main criteria: patterns of inheritance, axial level of the most anterior fusion, and common anomalies associated with KFS. KF1 exhibits a recessive inheritance pattern, C1-2 fusion, and a type I, II, or III fusion pattern. KF2 is autosomal dominant, C2-3 fusion, and a type I, II, or III pattern. KF3 is characterized by isolated fusions between cervical vertebrae (except C1-2) and exhibits a recessive or diminished penetrance inheritance pattern. Finally, patients with KF4 show signs of Wildervank and Duane syndromes [17]. 

A third classification system was introduced even more recently by Samartzis and colleagues to differentiate KFS patients further. In this system, type I patients present with a single cervical vertebra fusion, type II patients exhibit multiple noncontiguous fused segments, and type III patients demonstrate multiple contiguous fused cervical segments [18,19]. This system has already been used in practice to classify various blocked vertebral specimens [20]. 

Although KFS typically refers to the congenital fusion of cervical vertebrae, vertebral synostosis in the thoracic and lumbar spines has also been observed. In one population, the frequency of lumbar CVS was 0.75%. This included one L1-L2 fusion associated with kyphosis and two thoracolumbar (T12-L1) synostoses associated with kyphosis and lordosis. Also, this region's CVS was associated with ligaments' ossification and osteophytic growths extending from the margins of vertebral bodies [20].

The classic clinical triad of KFS includes a short neck, limited head and neck movements, and low posterior headlines. However, specific anomalies have been reported in patients with type III KFS, which involves synostosis of the thoracic and lumbar regions [21,22]. These anomalies can also include scoliosis, fused crossed renal ectopia, and various rib and craniofacial anomalies [22]. There can also be hypermobility of the interspace above the site of vertebral fusion, rendering these areas more susceptible to adjacent level disease and stenosis of the neural canal with associated neurological deficits due to alteration of spinal mechanics [21]. Clinicians need to be aware of these anomalies when diagnosing, characterizing, and treating patients with KFS. The authors sincerely thank those who donated their bodies to science so that anatomical research could be performed. Results from such research can potentially increase humankind's overall knowledge that can then improve patient care. Therefore, these donors and their families deserve our highest gratitude [23].

## Conclusions

CVS is a rare developmental condition resulting from the failure of vertebral resegmentation during fetal life. It is mostly found incidentally and can be associated with other congenital anomalies; however, it can represent underlying syndromic manifestations of Klippel Feil syndrome, which can progress to spinal deformity and spinal stenosis. Early diagnosis and thorough workup will help establish the diagnosis and document long-term changes caused by these conditions.

## References

[1] Kulkarni V, Ramesh BR (2012). A spectrum of vertebral synostosis. Int J Basic Appl Med Sci.

[2] Adler JT (2009). Gray's Anatomy: The Anatomical Basis of Clinical Practice, 40th Edition.

[3] Yochum TR, Rowe LJ (2007). Essentials of Skeletal Radiology, 3rd ed, 2 vols.

[4] Kumar R, Guinto FC Jr, Madewell JE, Swischuk LE, David R (1988). The vertebral body: radiographic configurations in various congenital and acquired disorders. Radiographics.

[5] Clarke RA, Singh S, McKenzie H, Kearsley JH, Yip MY (1995). Familial Klippel-Feil syndrome and paracentric inversion inv(8)(q22.2q23.3). Am J Hum Genet.

[6] Erdil H, Yildiz N, Cimen M (2003). Congenital fusion of cervical vertebrae and its clinical significance. J Anat Soc India.

[7] Kaplan KM, Spivak JM, Bendo JA (2005). Embryology of the spine and associated congenital abnormalities. Spine J.

[8] (2021). Victor’s Notes.Cranial and vertebral anomalies. http://www.neurosurgeryresident.net/Contents.htm.

[9] Colbjørn Larsen K, Fuchtbauer EM, Brand-Saberi B (2006). The neural tube is required to maintain primary segmentation in the sclerotome. Cells Tissues Organs.

[10] Noordeen MH, Garrido E, Tucker SK, Elsebaie HB (2009). The surgical treatment of congenital kyphosis. Spine (Phila Pa 1976).

[11] Sture JF (2021). Biocultural perspectives on birth defects in medieval urban and rural English populations.. http://etheses.dur.ac.uk/4138/.

[12] Chandraraj S (1987). Failure of articular process (zygaphophyseal) joint development as a cause of vertebral fusion (blocked vertebrae). J Anat.

[13] McMaster MJ, Singh H (1999). Natural history of congenital kyphosis and kyphoscoliosis. a study of one hundred and twelve patients. J Bone Jt Surg Am.

[14] Woo JY, Pee YH, An Y, Lim JH, Jang IT (2016). A case of acute compression fracture in block vertebra. Spine J.

[15] Kaur D, Billore N, Kumar G, Aggarwal P (2013). Chronic low back pain: block vertebra. BMJ Case Rep.

[16] Shankar VV, Kulkarni RR (2011). Block vertebra; fusion of axis with the third cervical vertebra - a case report. Int J Anat Varia.

[17] (1987). The essentials of skeletal radiology. https://www.abebooks.com/servlet/BookDetailsPL?bi=22540876648&searchurl=sortby%3D17%26tn%3Dessentials%2Bskeletal%2Bradiology&cm_sp=snippet-_-srp1-_-title3.

[18] Clarke R (2021). Klippel-Feil Syndrome - NORD (National Organization for Rare Disorders). https://rarediseases.org/rare-diseases/klippel-feil-syndrome/.

[19] Samartzis DD, Herman J, Lubicky JP, Shen FH (2006). Classification of congenitally fused cervical patterns in Klippel-Feil patients: epidemiology and role in the development of cervical spine-related symptoms. Spine (Phila Pa 1976).

[20] Senoglu M, Ozbag D, Gumusalan Y (2008). Two cases of Klippel-Feil syndrome. Int J Anat Var.

[21] Sar M, Behera S, Bara D (2018). Developmental abnormalities of vertebral column: a study in dried vertebrae of western Odisha population. J Anat Soc India.

[22] Elster AD (1989). Bertolotti's syndrome revisited. Transitional vertebrae of the lumbar spine. Spine (Phila Pa 1976).

[23] Naikmasur VG, Sattur AP, Kirty RN, Thakur AR (2011). Type III Klippel-Feil syndrome: case report and review of associated craniofacial anomalies. Odontology.

[24] Iwanaga J, Singh V, Ohtsuka A (2021). Acknowledging the use of human cadaveric tissues in research papers: recommendations from anatomical journal editors. Clin Anat.

